# S-3′-hydroxy-7′, 2′, 4′-trimethoxyisoxane, a novel ferroptosis inducer, promotes NSCLC cell death through inhibiting Nrf2/HO-1 signaling pathway

**DOI:** 10.3389/fphar.2022.973611

**Published:** 2022-08-29

**Authors:** Jing Chen, Songlin Zhou, Xian Zhang, Huange Zhao

**Affiliations:** ^1^ Key Laboratory of Tropical Translational Medicine of Ministry of Education, NHC Key Laboratory of Control of Tropical Diseases, School of Tropical Medicine, Hainan Medical University, Haikou, China; ^2^ Schools of Basic Medicine and Life Sciences, Hainan Medical University, Haikou, China

**Keywords:** S-3′-hydroxy-7′,2′,4′-trimethoxyisoxane, cell death, ferroptosis, Nrf2/HO-1 signaling pathway, non-small cell lung cancer

## Abstract

**Background:** Ferroptosis is a newly discovered and promising non-apoptotic programmed cell death (PCD), and inducing ferroptosis in cancer cells could open up a novel avenue for drug screening and cancer therapy. S-3′-hydroxy-7′, 2′, 4′-trimethoxyisoxane (ShtIX), a new isoflavane compound, has been reported to possess cytotoxicity in non-small cell lung cancer (NSCLC). The aim of this research is to explore the ShtIX-induced cell death form and its underlying molecular mechanism in NSCLC cells.

**Methods:** Cell proliferation, cell cycle arrest, and cell death tests were used to assess the ability of ShtIX to kill NSCLC cells. Iron metabolism, Fe^2+^ content, reactive oxygen species (ROS) production, lipid peroxide (MDA) level, glutathione (GSH) level, and glutathione peroxidase 4 (GPX4) level were used to determine ferroptosis caused by ShtIX. We employed western blot, quantitative real-time PCR, and Nrf2 interference in NSCLC cells to investigate the roles of Nrf2/HO-1 in ShtIX-induced ferroptosis. In a xenograft nude mouse model, the anticancer efficacy of ShtIX and the function of ferroptosis were studied.

**Results:** Our research shows that ShtIX can selectively kill NSCLC cells while sparing normal cells and that ShtIX-induced cell death can be efficiently reversed by the ferroptosis inhibitors and the iron chelator, but not by other cell death inhibitors. After cells were treated with ShtIX, there was an increase in Fe^2+^ content and lipid peroxidation accumulation, as well as a drop in GSH and GPX4 levels, all of which are indicators of ferroptosis. ShtIX also reduced the expression of Nrf2 and HO-1, and genetic Nrf2 silencing in NSCLC enhanced the effect of ShtIX-induced ferroptosis. Additionally, ShtIX retards tumor growth and induced ferroptosis through Nrf2/HO-1 signal pathway in the A549 xenograft model, whereas Fer-1 lessens the anticancer effect.

**Conclusion:** This work provided the evidence that ShtIX caused ferroptosis in NSCLC cells, and inhibiting the Nrf2/HO-1 pathway can considerably exacerbate the effect of ShtIX-induced ferroptosis. The study establishes ShtIX as a promising natural ferroptosis inducer for the treatment of NSCLC.

## Introduction

**FIGURE 1:**
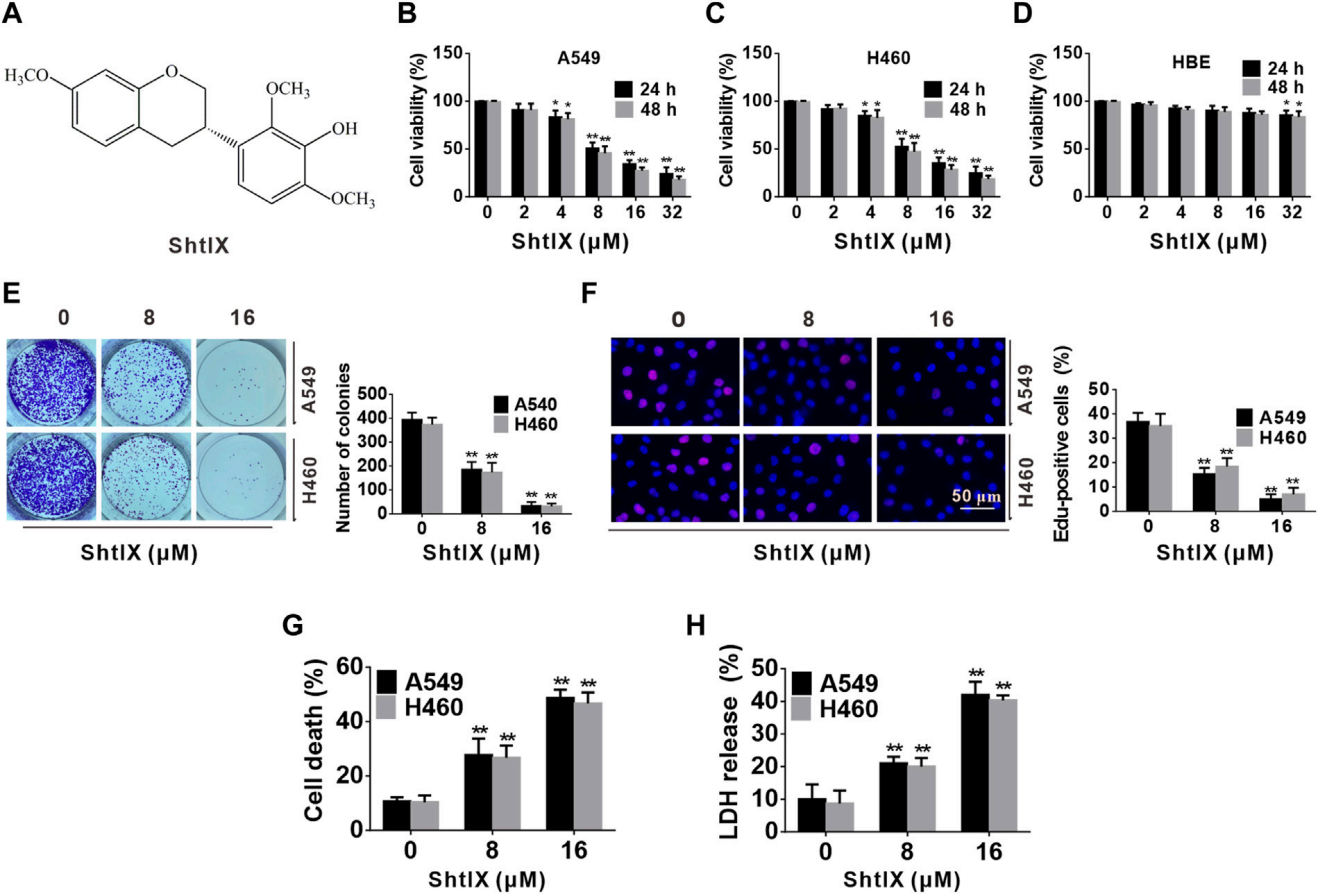
ShtIX inhibits cell proliferation and causes cell death in NSCLC cells. **(A)** The molecular structure of ShtIX. **(B,C)** Cell viability was measured by CCK-8 assay after cells were exposed to indicate concentrations of ShtIX at different times. **(D)** The viability of normal lung epithelial HBE cells was determined by CCK-8 assay. **(E)** The ability of cells to form colonies was observed after cells treated with indicated dose of ShtIX. **(F)** Cells proliferation was detected by EdU assay. **(G,H)** The NSCLC cell death was analyzed by trypan blue exclusion and LDH release assay. Data were presented as the mean ± SD of 3 independent experiments. *p < 0.05, **p < 0.01.

Bioactive compounds derived from medicinal plants have long been acknowledged as a valuable source for medication development, and many of them have potential applications in cancer treatment (Desai et al., 2008). A novel isoflavane, S-3′-hydroxy-7′, 2′, 4′-trimethoxyisoxane (ShtIX, Figure 1A), was obtained from the heartwood of *Dalbergia odorifera* T. Chen (Chinese called Jiangxiang) (Zhu et al., 2020). In South China, the heartwood of *D. odorifera* is used to treat a wide range of maladies, including blood abnormalities, cardiovascular diseases, ischemia, and pain relief (Meng et al., 2019). Sesquiterpenes, flavonoids, and other phenolic chemicals have been identified as the main components of *D. odorifera* in previous phytochemical studies (Zhao et al., 2000). Isoflavane, a subclass of isoflavonoids, has been shown to exhibit anti-proliferative effects against various types of cancer cell lines and cause apoptosis *via* different molecular mechanisms. (Hsu et al., 2011; Tsai et al., 2011; Jiang et al., 2016; Kaennakam et al., 2017; Wang et al., 2018; Peng et al., 2020; Castillo-Bautista et al., 2021; Wang et al., 2021). In this study, ShtIX was found to have stronger cytotoxicity against NSCLC cells than other cancer cells, as we were able to show here. However, the ShtIX-induced cell death mode and its underlying molecular mechanism require more research.

In the last decade, apoptosis, autophagy, ferroptosis, pyroptosis, necroptosis, NETosis, alkaliptosis, and other forms of PCD have all been discovered. However, anticancer medication research has mostly focused on apoptosis for a long time. Since apoptotic escape of tumor cells is one of the primary mechanisms responsible for multidrug resistance, other cell death types have received increasing attention from researchers (Indran et al., 2011; Giussani et al., 2014). Ferroptosis is a recently identified and promising non-apoptotic PCD pattern named by Scott J. Dixon in 2012, is distinct from other PCD forms (such as apoptosis, necroptosis, and autophagy) in morphology, biochemistry, and gene expression (Dixon et al., 2012; Yu et al., 2017). The buildup of iron-dependent and lipid peroxidation, which cause oxidative stress and cell death, is the typical symptom of ferroptosis. Ferroptotic cells have specific morphological alterations, such as an increased in the density of the mitochondrial membrane, a reduced or disappeared mitochondrial ridge, and a ruptured outer mitochondrial membrane (Jiang et al., 2021). According to recent research, ferroptosis is connected to a number of physiological and pathological processes in the body, and cancer cells are particularly susceptible to it because of the phenomenon of “iron addiction” (Toyokuni, 2019). There is growing evidence that ferroptosis can be harnessed for cancer treatment, and the use of ferroptosis to develop novel anticancer strategies has recently attracted increasing research attention (Xu et al., 2019; Jiang et al., 2020). So far, it has been noted that a number of first-line chemotherapy medications, including sorafenib, sulfasalazine, and artesunate, have been reported to promote ferroptotic cell death both *in vitro* and *in vivo* (Dai et al., 2020). Therefore, induction of ferroptosis in cancer cells could open up a novel way for both drug screening and cancer therapy.

Nuclear factor E2 related factor 2 (Nrf2) is a transcription factor that activates endogenous antioxidant response elements and is a critical regulator for preserving cellular redox homeostasis (Sajadimajd and Khazaei, 2018). The Nrf2 transcription factor regulates a large number of genes, many of which are directly or indirectly involved in the modulated of ferroptosis (Kerins and Ooi, 2018; Dodson et al., 2019; Anandhan et al., 2020). Heme oxygenase-1 (HO-1) is the main downstream target protein of Nrf2 in oxidative stress. According to new studies, Nrf2 and HO-1 are the important mediators of the ferroptotic response (Jiang et al., 2020b; Ma et al., 2020; Li et al., 2021; Lou et al., 2021; Wei et al., 2021). In the present study, we demonstrated that ShtIX triggered ferroptosis in NSCLC cells by inhibiting the Nrf2/HO-1 signaling pathway. The findings provided evidence that the ShtIX is a promising naturally derived ferroptosis inducer that can be used to create a new treatment strategy for lung cancer.

## Materials and methods

### Plant materials and isolation of S-3′-hydroxy-7′, 2′, 4′-trimethoxyisoxane

The heartwood of *D. odorifera* T. Chen was purchased from Haikou, Hainan Province, China, in September 2020. The specimen was authenticated by Professor Jun Wang, and the voucher specimen was numbered as AN202009. SthIX was extracted as previously reported (Zhu et al., 2020). The pure extract was diluted in DMSO at a stock concentration (1 mM) for the following experiments.

### Cell lines and cell cultures

Human gastric cancer cell line SGC-7901, human lung adenocarcinoma cell lines A549 and H460, human colorectal cancer cell line SW480, human liver cancer cell line BEL-7402, human breast cancer cell line MCF-7, human cervical cancer cell line Hela, and human lung epithelial cell line HBE were obtained from Shanghai Institute of Cell Biology, Chinese Academy of Sciences (Shanghai, China). All cells were cultured with RK Memorial Institute 1,640 (RPMI1640) medium (Gibco, United States).

### Cytotoxic activity assay

The cytotoxicity activity of ShtIX against all tested cancer cell lines and normal cells was investigated using the Cell Counting Kit-8 (CCK-8) (Beyotime, Shanghai, China) in accordance with operating instructions. IC_50_ values of the treated cancer and normal cells were calculated using GraphPad Prism (GraphPad, San Diego, CA). A549 cells were the most sensitive to ShtIX compared to other cancer cell lines after 24 h treatment. For subsequent research, human lung cancer cell lines (A549 and H460) were employed.

### Cell proliferation assay

To detect the effects of ShtIX on NSCLC cell proliferation, we first used cell clone formation experiments to observe the single cells to form a colony. Briefly, 5×10^2^ cells were seeded in a 6-well cell culture plate. After being inoculated with the corresponding dose of ShtIX, the cells were grown for 12 days (changing the culture medium containing ShtIX every 3 days). Then, the cells were fixed, stained, counted, and photographed. After that, we used the EdU-594 cell proliferation detection kit (Beyotime, Shanghai, China) to assess the DNA synthesis following the manufacturer’s instructions. Cell nuclei were counterstained with DAPI (Sigma-Aldrich, St Louis, United States). The cells incorporating EdU were monitored under a fluorescence microscope (Olympus Corporation, Tokyo, Japan).

### Cell death assay

Cell death was determined using trypan blue staining assay (Beyotime, Shanghai, China) and lactate dehydrogenase (LDH) release analysis (Sigma-Aldrich, MO, United States). Trypan blue will penetrate dead cells but will not stain living ones. LDH is the most frequently utilized marker in death/cytotoxicity assay. Trypan blue staining and LDH release assay were carried out according to the manufacturer’s instructions.

### Cell death inhibition assay

Caspase inhibitor Z-VAD-FMK (Sigma-Aldrich, United States), the autophagy inhibitor 3-Methyladenine (3-MA, Sigma-Aldrich, United States), the necroptosis inhibitorNecrostatin-1 (Nec-1,Sigma-Aldrich, United States), the ferroptosis inhibitor Ferrostatin-1 (Fer-1, Sigma-Aldrich, United States) and deferoxamine (DFO, Sigma-Aldrich, United States) were used in inhibitor investigations.

### Cell cycle assay

The percentages of cells in each phases of the cell cycle (G0/G1, S, and G2/M) were quantified by flow cytometry. The experiments’ procedures have already been described (Zhang et al., 2022).

### Cell apoptotic events analysis

To determine the molecular mechanism of ShtIX-induced cell death, apoptosis, the classical mode of PCD, was studied. Hoechst 33,258 staining was used to observe the morphological alterations in the cells. Flow cytometry was employed to detect apoptosis, and western blot was used to analyze the expression of apoptosis-related protein. Detailed operating methods and steps were conducted as previously mentioned (Zhao et al., 2021).

### Transmission electron microscope assay

Cells were cultured overnight and then treated with indicated dosage of ShtIX for 24 h. Cells were digested, collected, and fixed with 2.5% glutaraldehyde and 1% osmium tetroxide. Then, cells are using various grades dehydrated graded ethyl alcohols (50–100%) and embedded with epoxy resin. Subsequently, cell samples were cut into ultrathin sections and stained with 2% uranyl acetate and lead citrate. The images were acquired under a transmission electron microscope (HT7700, Hitachi, Tokyo, Japan).

### ROS and lipid ROS assay

The ROS generation was determined by the ROS detection kit (Beyotime, Shanghai, China). Lipid ROS level was examined by C11-BODIPY581/591 (Thermo Scientific, United States) as a probe. Briefly, A549 and H460 cells were seeded in a 6 well cell culture plate and exposed to16 μM ShtIX with or without Fer-1 for 24 h. Cells were digested, harvested, and suspended in a final concentration of 10 μM DCFH-DA and C11-BODIPY581/591 in the dark for 20 min. After that, cells were treated 3 times with serum-free media in order to eliminate non-intracellular DCFH-DA and C11-BODIPY581/591 completely. Intracellular ROS and lipid ROS were measured by flow cytometry (BD Accuri C6, United States).

### Ferrous iron assay, GSH and MDA assay

Cells seeded in a 6 well culture plate were exposed to different concentrations of ShtIX. The ferrous iron assay (Thermo Scientific, Waltham, United States), GSH assay (Beyotime, Shanghai, China) and MDA (Thermo Scientific, Waltham, United States) assay were carried out using the iron assay kit, the GSH assay kit and MDA assay kit, respectively, in accordance with the manufacturer’s protocol.

### RNA isolation and quantitative real-time polymerase chain reaction

Total RNA was obtained from A549 and H460 cells using TRIzol^®^ Reagent (Thermo Fisher Scientific, Waltham, United States). The extracted RNA was quantified and purified and was reversed into cDNA using the Prime ScriptTM RT Reagent Kit with gDNA Eraser (TaKaRa, Beijing, China). RT PCR was performed using SYBR Premix Ex Taq II (TaKaRa, Beijing, China) reagent by the Bio-Rad qPCR system (Bio-Rad, Hercules, CA). Nrf2 (forward prime: 5′-AGT​CCT​GGT​CAT​CGG​AAA​AC-3′, reverse prime: 5′-ATG​GAG​AGC​TTT​TGC​CCT​AA-3′). HO-1 (forward prime: 5′-CTC​TTG​GCT​GGC​TTC​CTT​AC-3′, reverse prime: 5′-TCC​TTC​CTC​CTT​TCC​AGA​GA-3′). *β*-actin (forward prime: 5′-CCA​CGA​AAC​TAC​CTT​CAA​CTC​C-3′, reverse prime: 5′-GTG​ATC​TCC​TTC​TGC​ATC​CTG​T-3′). All primers were synthesized by Sangon Biotech (Shanghai, China). Each experiment was performed 3 times. *β*-actin was employed as an internal reference.

### RNA interference and overexpression experiment

For silencing the Nrf2 gene, NSCLC cells were incubated in 6-well plates overnight. Then, cells were transfected with 20 μM Nrf2 siRNA or si control siRNA (Hanbio, Shanghai, China) using Lipofectamine 3000 (Invitrogen, Carlsbad, CA, United States) in Opti-MEM medium (Gibco BRL, Grand Island, NY, United States). Briefly, dilute 8.4 ng siRNA into 100 μL Opti-MEM and mix thoroughly. Add 2 μL of RNA Fit to the 100 μL siRNA medium and shake for 10 s. After that, incubate to form a complex at room temperature for 15 min between siRNA and RNA Fit. While gently swirling the plate, drop the siRNA mixture into the 6-well plate. After transfection, a western blot was used to confirm the effectiveness of the silencing. For the overexpression experiment, Lipofectamine^®^ LTX Reagent (Invitrogen, Carlsbad, CA, United States) was used to transfecte A549 and H460 cells with a final concentration of 0.5 ng/μL pcDNA3-human Nrf2 or pcDNA3 vector. The medium was switched out for new medium after 24 h, and cell survival rate data and ferroptosis were measured after 48 h.

### *In vivo* experiments

The procedure for establishing xenograft nude mice was followed as previously described (Zhao et al., 2021). When tumor volumes in xenograft nude mice reached an average of roughly 100 mm^3^, the mice were randomly divided into 3 groups of 6 mice each: control, ShtIX, and ShtIX + Fer-1. The treated group received ShtIX or ShtIX combined with Fer-1 injections into the tail vein of the mice every three days for 7 times, whereas the control group received saline. Every four days, the volume and weight of the tumors were measured. As soon as the test was completed, the nude mice were slaughtered, and the tumor tissues were retrieved. The *in vivo* experiments were approved by the Animal Care and Use Committee of Hainan Medical College and following the animal rules.

### Western blot assay

The western blot assay was carried out as previously described (Zhao et al., 2021). The primary antibodies against caspase-3 (Abcam, ab65080), cyclin D1 (Abcam, ab226977), CDK4 (Abcam, ab137675), p21 (Abcam, ab227443), FPN (Abcam, ab235166), TF (Abcam, ab84036), FTH1 (Abcam, ab65080), GPX4 (Abcam, ab125066), Nrf2 (Abcam, ab137550), HO-1 (Abcam, ab13243) and *β*-actin (Abcam, ab8227) were used in this investigation.

### Statistical analysis

The experimental data were analyzed using the GraphPad Prism software in this study. All data are presented as mean ± SD of at least 3 separate experiments. Student’s t-test is used to compare statistical differences between two groups, while one/two-way ANOVA is used to assess statistical data differences among different groups. p*< 0.05, p**< 0.01, *p* ^#^< 0.05 and *p* ^##^< 0.01 were consider to be statistically significant different in all analyses.

## Results

### ShtIX exhibits anti-proliferation activities on selected cancer cell lines

The cell viability of ShtIX on selected cancer cell lines is first assessed using the CCK-8 assay. A549 cells were the most sensitive to ShtIX (IC_50_ = 7.9 ± 0.52 μM) compared to other selected cancer cell lines (Table 1). Therefore, NSCLC cell lines (A549 and H460 cells) were chosen for the subsequent experiments.

**TABLE 1:** IC50 values of ShtlX on selected cancer cell line.

Types of cell lines	SGC-7901	A549	Hela	BEL-7402	SW480	MCF-7
IC_50_ (µM)	21.5 + 0.15	7.9 ± 0.52	18.9 ± 0.31	36.9 + 0.93	40.5 + 1.16	61.25 + 8.25

To further determine the cytotoxicity of ShtIX on NSCLC cells, a CCK-8 test was employed to detect the cell viability of NSCLC and normal lung epithelial cell HBE after cells were exposed to various concentrations (0, 2, 4, 8, 16, 32 μM) of ShtIX for 24 and 48 h. As illustrated in Figures 1B–D, A549 and H460 cells viability were significantly reduced in a dose and time-dependent manner after cells were exposed to ShtIX, but normal cells showed minimal toxicity. Next, the effect of ShtIX on cell proliferation was examined by using the colony formation assay and EdU staining. The results from colony formation indicated that ShtIX markedly suppressed NSCLC cells proliferation in a concentration-dependent manner (Figure 1E). After cells were treated with ShtIX for 24 h, there were less EdU-positive cells in NSCLC cells (Figure 1F). Additionally, trypan blue staining and intracellular LDH release were used to assess how ShtIX affects cell death. As illustrated in Figures 1G, H, ShtIX promoted cell death and intracellular LDH release in NSCLC cells with increasing concentrations. According to the findings, ShtIX not only inhibited cell proliferation but also led to cell death in NSCLC cells.

### ShtIX causes cell cycle arrest in NSCLC cells

**FIGURE 2:**
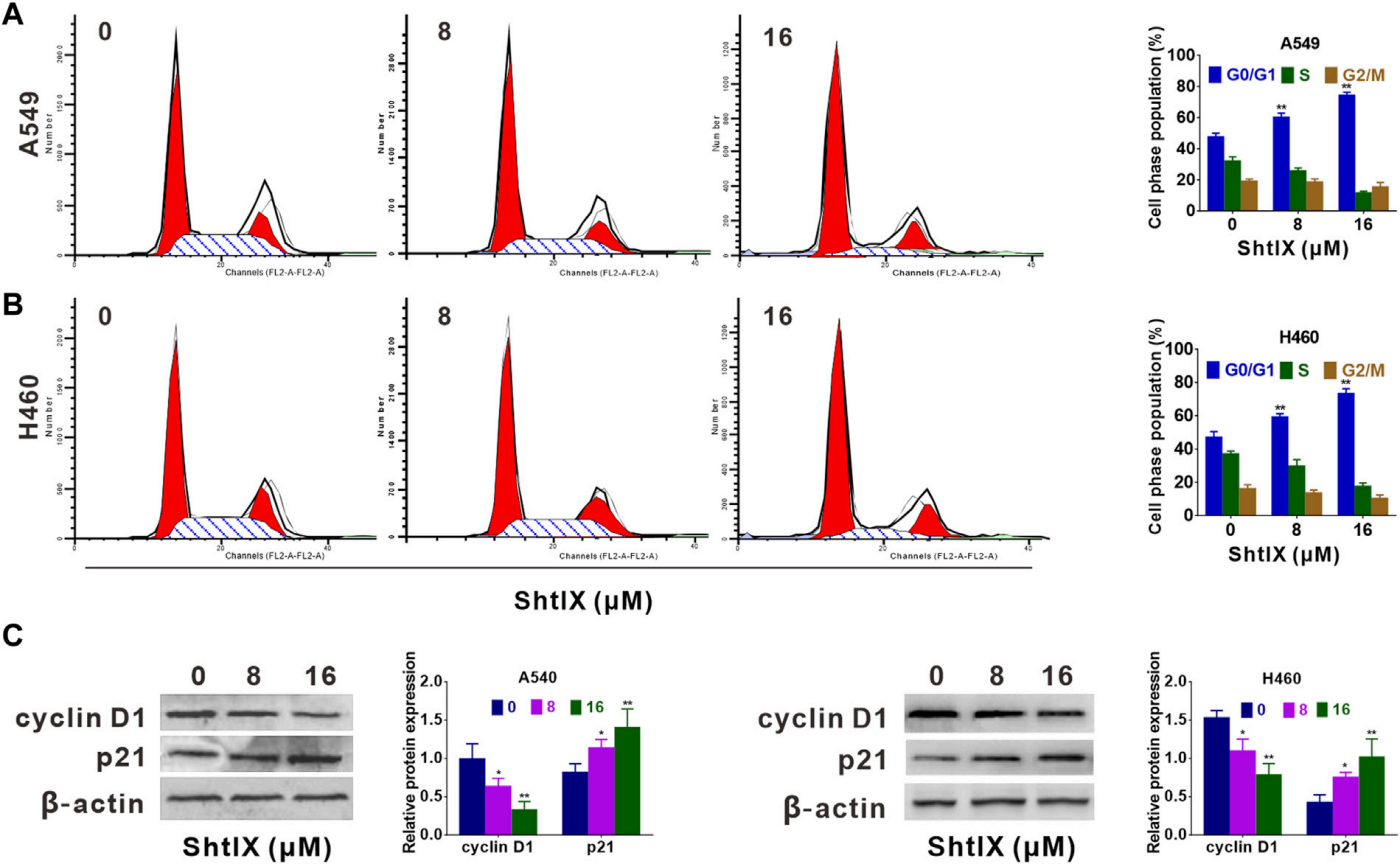
ShtIX inhibited cell cycle progression in NSCLC cells. Cells were exposed to specified concentrations of ShtIX for 24 h, and then were analyzed by PI staining. **(A,B)** DNA content was analyzed by flow cytometry. **(C)** Cell cycle related-proteins were determined by western blot. Data were presented as the mean ± SD of three independent experiments. *p < 0.05, **p < 0.01.

In order to elicit the mechanisms of ShtIX anti-proliferation in NSCLC cells, the cell cycle progression was observed using a PI staining assay. After cells treatment with indicated dosage of ShtIX for 24 h, the G0/G1 percentage of both A549 and H460 cells considerably increased, whereas the S and G2/M proportion decreased (Figures 2A,B). Furthermore, the expression of proteins related to the G0/G1 phase, including cyclin D1, CDK4 and p27 in the G0/G1 phase was analyzed by western blotting. As illustrated in Figure 2C, cyclin D1 and CDK4 expression were decreased in ShtIX-treated cells whereas p27 expression was increased. These findings demonstrated that cell cycle arrest in the G0/G1 phase contribute to ShtIX anti-proliferation in NSCLC cells.

### Apoptosis is not the primary cell death mode induced by ShtIX in NSCLC cells

**FIGURE 3:**
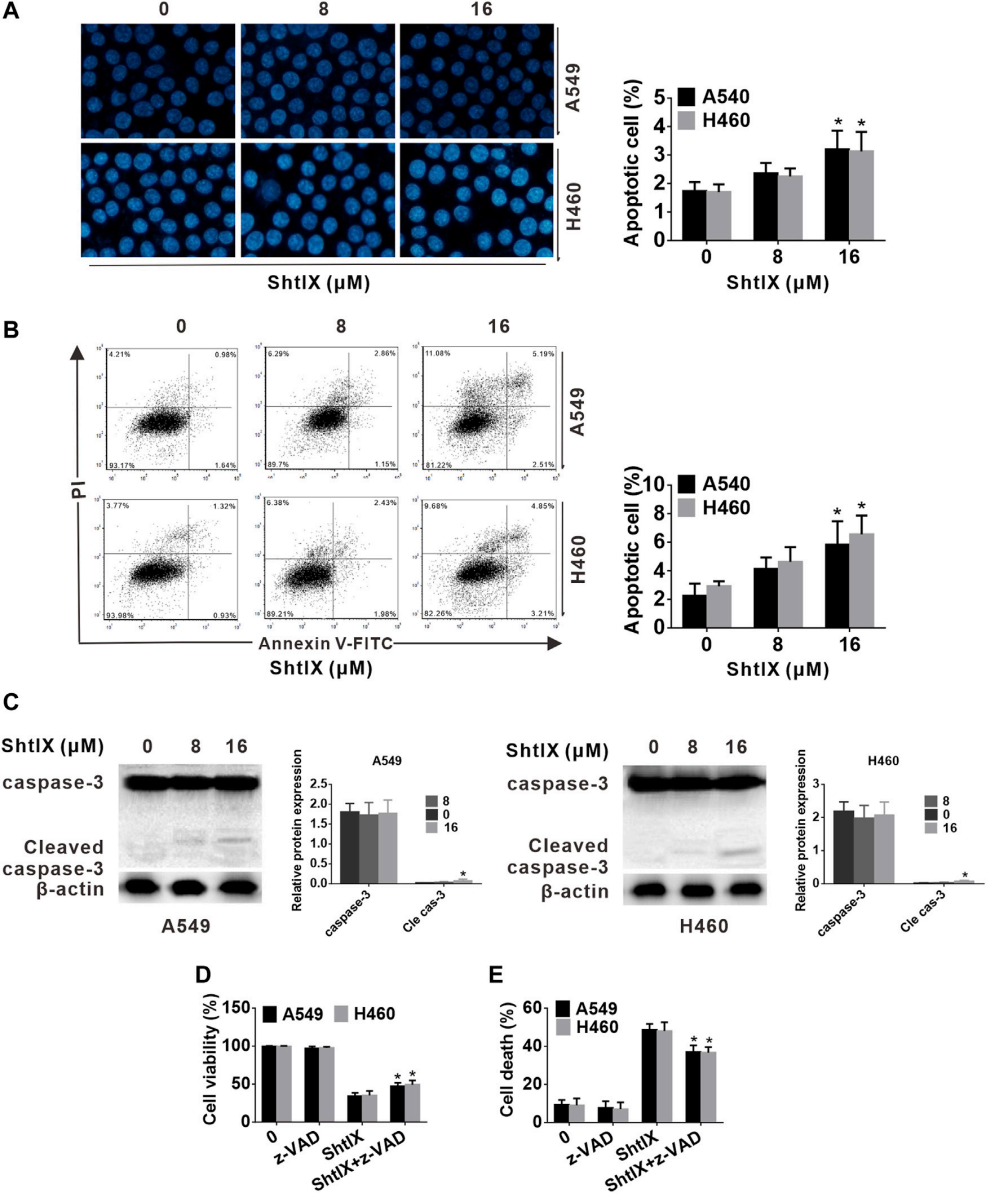
The apoptotic events of ShtIX-induced were observed. Cells were treated with the given dose of ShtIX for 24 h **(A)** NSCLC cells morphological change was observed by Hoechst 33,258 staining. **(B)** Early and late apoptotic cells induced by ShtIX were detected by Annexin V-FITC/PI. **(C)** The apoptotic related-proteins were analyzed by western blot. **(D)** Cells were treated with ShtIX in combination with or without Z-VDA-FMK, the cell viability was detected using CCK-8 assays. **(E)** Cells were treated as in D, the cell death were detected by LDH release assay. Data are expressed as mean ± SD of triplicate experiment. *p < 0.05, **p < 0.01.

To confirm the ShtIX-induced cell death manner in NSCLC cells, apoptosis-related markers were first examined. Evidence from apoptotic morphological experiments showed that cells treated with 8 μM ShtIX did not exhibit the typical apoptotic morphological alterations, such as shrinkage, nuclear fragmentation and chromatin condensation. Only when ShtIX was present in a concentration of 16 μM did the cell apoptotic morphology change (Figure 3A). Likewise, the results from Annexin V/PIstaining displayed that the number of apoptotic cells did not rise after cells were exposed to 8 μM ShtIX, and only rose at 16 μM ShtIX (Figure 3B). Furthermore, the cleaved form of caspase-3 was also active at a dose of 16 μM in NSCLC cells (Figure 3C). Under the same conditions, Z-VAD-FMK reversed ShtIX (16 μM)-induced NSCLC cell death, but had no effect on 8 μM ShtIX (Figures 3D,E). Based on the above results, it is reasonable to conclude that there might be other pattern of cell death induced by ShtIX in NSCLC cells.

### ShtIX induces NSCLC cell death is associated with iron metabolism

**FIGURE 4:**
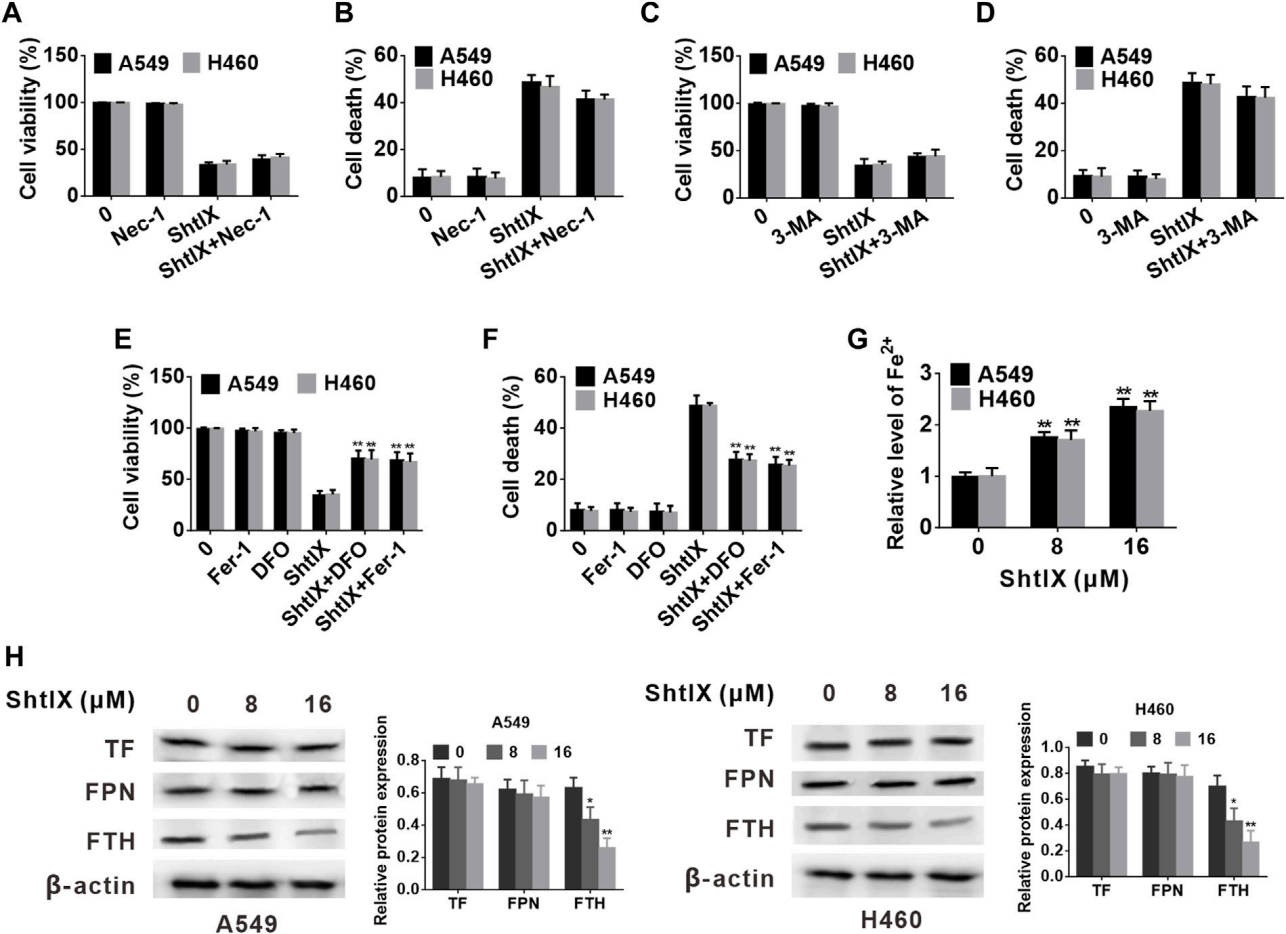
ShtIX induces NSCLC cell death is associated to iron metabolism. **(A,B)** A549 and H460 cells viability and cell death were determined after being treated with 16 μM ShtIX in the presence or absence of Nec-1 **(C,D)** A549 and H460 cells viability and cell death were determined after being treated with 16 μM ShtIX in the presence or absence of 3-MA. **(E,F)** A549 and H460 cells viability and cell death were determined after being treated with 16 μM ShtIX in the presence or absence of DFO or Fer-1. **(G)** Fe^2+^ content in A549 and H460 cells was observed after cells were treated with indicated dose of ShtIX for 24 h, **(H)** Iron homeostasis-related proteins were evaluated by western blot. Data are expressed as mean ± SD of triplicate experiment. *p < 0.05, **p < 0.01.

In order to determine the pattern of cell death that was caused in A549 and H460 cells, the effects of various cell death inhibitors conjunction with ShtIX were assessed. As illustrated in Figures 4A–D, the autophagy inhibitor 3-MA and the necroptosis inhibitor Nec-1 had no discernible effects on the ShtIX-induced NSCLC cell death. Interestingly, the ferroptosis inhibitor Fer-1 and the iron chelator DFO dramatically reversed the cell death caused by ShtIX compared to other inhibitors (Figures 4E,F). These findings imply that ShtIX’s anticancer properties depend on iron ions and that ShtIX-induced iron buildup may be a mechanism for ShtIX-induced cell death in NSCLC cells.

Iron ions are the prerequisite for the occurrence of ferroptosis, and excess of Fe^2+^ contributes to the generation of lipid ROS and induces ferroptosis. To explore whether ShtIX-induced cell death is associated with ferroptosis, the intracellular Fe^2+^ level in A549 and H460 cells was initially detected. As shown in Figure 4G, the intracellular Fe^2+^ level was apparently increased with an increasing dose of ShtIX. The outcomes demonstrate that the pattern of cell death brought on by ShtIX was an iron-dependent cell death mode. Additionally, to ascertain whether iron metabolism is connected to ShtIX-induced cell death in NSCLS cells, the expression of critical regulators of iron homeostasis, including ferritin heavy chain (FTH), ferroportin (FPN) and transferrin (TF) were evaluated. Here, we found that ShtIX dramatically reduced the expression of FTH1 with increasing concentration (Figure 4H), whereas the expression of TF and FPN was found have no noticeable changes. The resuts suggest that ShtIX altered iron homeostasis through regulating FTH to trigger cell death in NSCLC.

### ShtIX induces ferroptosis in NSCLC cells

**FIGURE 5:**
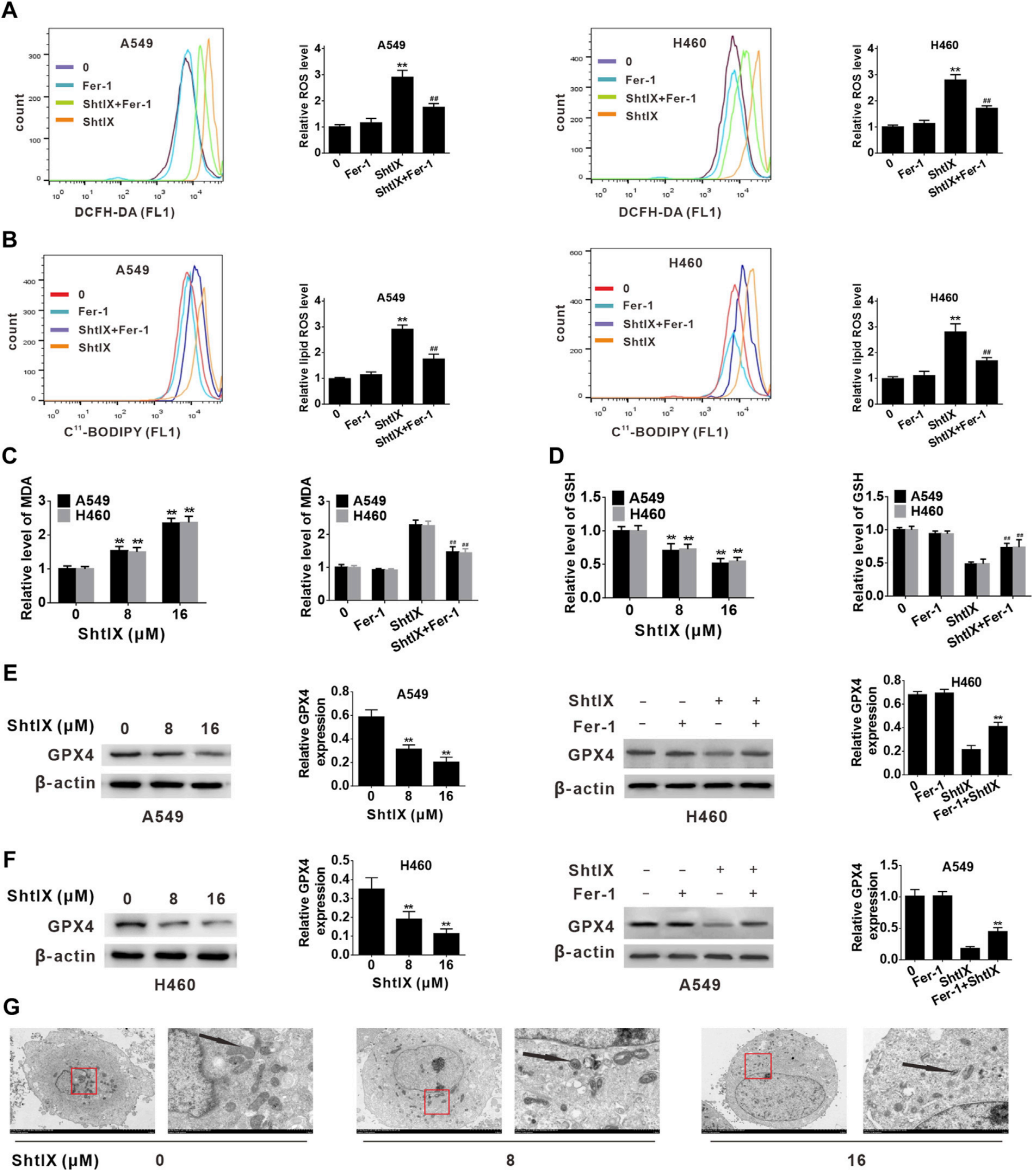
ShtIX induces ferroptosis in NSCLC cells. **(A)** A549 and H460 cells were treated with 16 μM ShtIX or ShtIX combined with Fer-1 for 24 h, and the level of ROS was detected by flow cytometry. **(B)** Cells were treated as **(A)**, then stained with BODIPY™ 581/591 C11 dye and lipid peroxidation was observed by flow cytometry. **(C)** Cells were treated with 8 and 16 μM ShtIX or 16 μM ShtIX combined with Fer-1 for 24 h, the MDA level was measured. **(D)** Cells were treated as **(C)**, the GSG level was measured. **(E,F)** The expression of GPX4 was analyzed by western blot. **(G)** Transmission electron microscopy images of A549 and H460 cells were treated with indicated dose of ShtIX. Scale bars: 5.0 µm; 1.0 µm. Data are expressed as mean ± SD of triplicate experiment. *p < 0.05, **p < 0.01, ^**#**^ *p* < 0.05, ^**##**^ *p* < 0.01.

Ferroptosis is likewise dependent on the production of ROS and the accumulation of lipid peroxides triggered by Fenton reactions, in addition to its dependence on iron. Here, we measured the ROS generation after treating NSCLC cells with ShtIX. As shown in Figure 5A, ShtIX-treated cells produced more ROS in NSCLC cells, whereas Fer-1 effectively reversed ROS accumulation in NSCLC cells. Next, we performed a flow cytometric study to measured the amounts of lipid ROS using the peroxidation-sensitive dye C11-BODIPY 581/591. As shown in Figure 5B, ShtIX treatment promoted the accumulation of lipid peroxidation in BODIPY-loaded cells, while Fer-1 can counteract this effect. Similar outcomes were observed for changes in the level of MDA in NSCLC cells (Figure 5C). The aforementioned findings suggest that ShtIX can trigger lipid peroxidation, a classic marker of ferroptosis. GSH is an antioxidant that scavenges lipid ROS through action of GPX4. Decreased the levels of intracellular GSH and GPX4 can lead to excessive oxidative stress and the occurrence of ferroptosis. As expected, intracellular GSH levels were markedly reduced after treatment with ShtIX, but Fer-1was able to restore them (Figure 5D). Likewise, the expression of GPX4 also decreased with increasing ShtIX concentration, which was offset by Fer-1 (Figures 5E,F). Furthermore, the typical morphological features of ferroptosis such as cell membrane rupture, mitochondrial shrinkage, thickening of the mitochondrial membrane density, and diminished or disappeared mitochondrial ridges, were observed in cell treated with ShtIX (Figure 5G). Together with above findings supports the idea that ShtIX causes ferroptosis in NSCLC cells.

### Nrf2/HO-1 plays a role in ShtIX-induced ferroptosis in NSCLC cells

**FIGURE 6:**
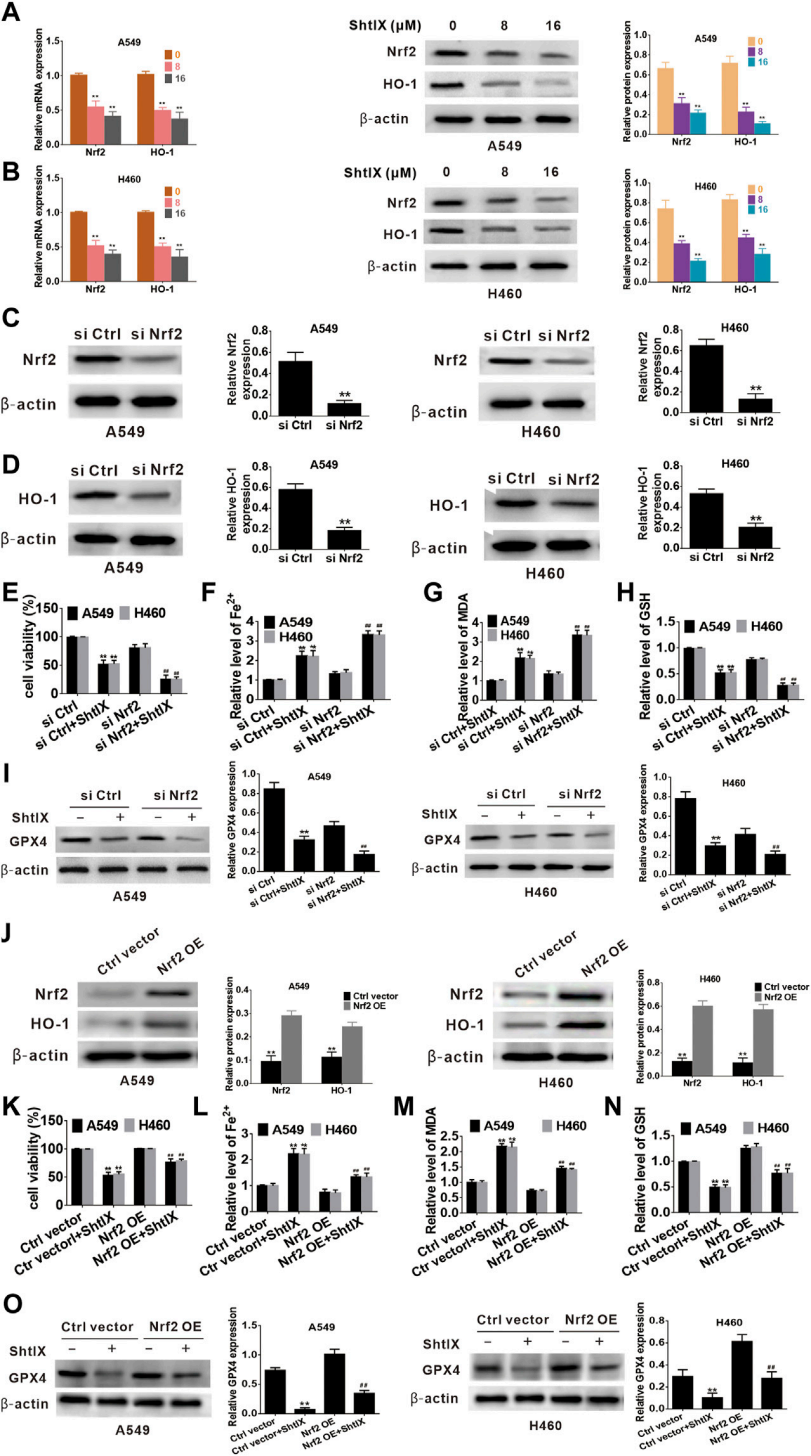
Nrf2/HO-1 plays a role in ShtIX-induced ferroptosis in NSCLC cells. **(A,B)** Cells treated with different dose of ShtIX for 24 h, the levels of Nrf2 and HO-1 were measured using RT-qPCR assay and western blot. **(C)** A549 and H460 cells were transfected with Nrf2 siRNA and control siRNA, and the efficiency of the knockdown Nrf2 was detected by western blot. **(D)** HO-1 expression in Nrf2 knockdown cells was assessed by western blot. **(E–I)** Cells treated as **(C)**, cell viability and ferroptosis-related markers were determined. **(J)** Western blot analysis was performed to detect the expression levels of Nrf2 and HO-1 protein following transfection with overexpression plasmids. **(K–O)** After cells were transfected with overexpression plasmid, cell viability and ferroptosis-related markers were evaluated. Data are expressed as mean ± SD of triplicate experiment.*p < 0.05, **p < 0.01, ^**#**^ *p* < 0.05, ^**##**^ *p* < 0.01.

Nrf2 serves as a key antioxidant regulatory transcription factor that can prevent ferroptosis-related cell death by activating both Nrf2 and its target genes (such as HO-1). Increasing evidence points to the critical role played by the Nrf2/HO-1 axis in mediating ferroptotic cell death. (Jiang et al., 2020b; Ma et al., 2020; Li et al., 2021; Lou et al., 2021; Wei et al., 2021). To clarify whether the Nrf2/HO-1 axis is involved in ShtIX-induced ferroptosis in NSCLC cells, the levels of Nrf2 and HO-1 were initially investigated by qRT-PCR and western blot. According to the results, ShtIX is able to dose-dependently decline the mRNA level and protein expression of Nrf2 and HO-1 in NSCLC cells (Figures 6A,B). To further highlight the role of Nrf2/HO-1 axis in ShtIX-triggered ferroptosis, we used RNA interference to knock down the Nrf2 gene in A549 and H460 cells. The effectiveness of siNrf2 was assessed by western blot analysis. As depicted in Figure 6C, following siNrf2 transfected, Nrf2 expression was markedly downregulated compared to the siRNA control, and there was a concomitant decreased of HO-1 level (Figure 6D). In the context of ShtIX treatment, cell viability was reduced after Nrf2 siRNA was transfected into NSCLC cells compared to the siRNA control (Figure 6E). Meanwhile, knockdown of Nrf2 expression led to an increased in the content of Fe^2+^ (Figure 6F) and MDA (Figure 6G), while GSH and GPX4 levels decreased (Figures 6H,I). Conversely, overexpression of Nrf2 reversed the downregulated the level of Nrf2 and HO-1 protein expression (Figure 6J) and decreased cell viability (Figure 6K) induced by ShtIX, and rescued the effects of ShtIX on ferroptosis (Figures 6L–O). Together, these observations imply that inhibited Nrf2/HO-1 pathway exacerbated ShtIX-induced ferroptosis in NSCLC cells.

### ShtIX retards NSCLC tumor growth and induced ferroptosis *in vivo*

**FIGURE 7:**
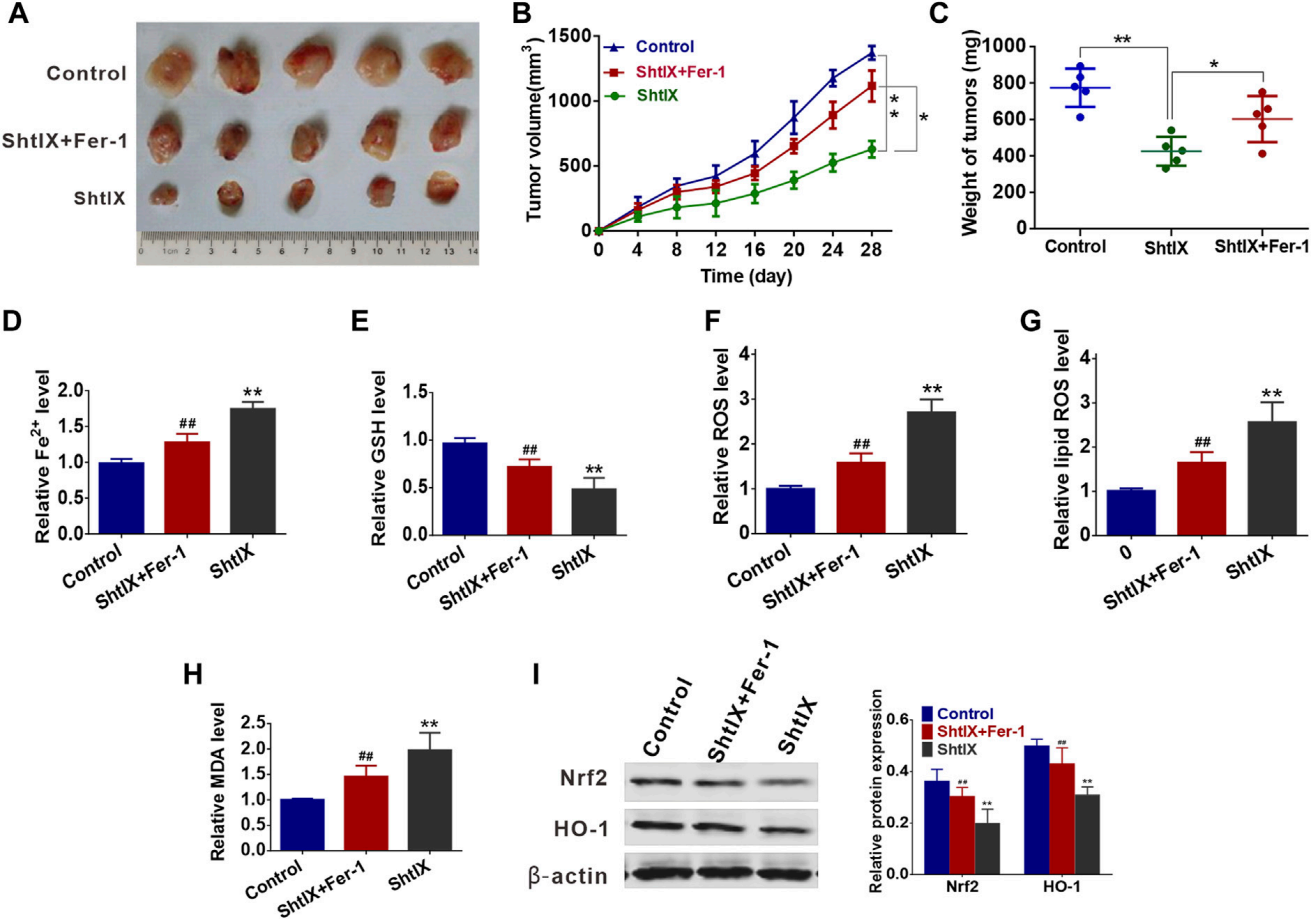
ShtIX retards tumor growth and induced ferroptosis *in vivo*. **(A)** The images of tumors dissected from mice. **(B)** Xenograft tumor volumes were detected every 3 days for 28 days. **(C)** Xenograft tumor weights were measured. **(D–I)** The levels of Fe^2+^, GSH, ROS, lipid ROS, MDA, Nrf2 and HO-1 in tumor tissues were analyzed. *p < 0.05, **p < 0.01, ^**#**^ *p* < 0.05, ^**##**^ *p* < 0.01.

A549 tumor cell xenograft method in BALB/c nude mice was created to examine the efficacy of ShtIX in reducing tumor growth and the function of ferroptosis in ShtIX-induced cell death *in vivo.* As shown in Figures 7A–C, ShtIX retards tumor growth, and tumor weight and volume were significantly lower in treated groups than those in the control group. On the other hand, ShtIX’s anticancer effects were lessened by Fer-1. Furthermore, ShtIX increased in Fe^2+^, ROS, lipid ROS, and MDA levels while decreasing GSH levels. These effects were reversed by co-treatment with Fer-1 (Figures 7D–H). Similar to what it did *in vitro*, ShtIX treatment decreased Nrf2 and HO-1 expression, while Fer-1 restored it (Figure 7I). These findings support the idea that ShtIX promot ferroptosis in NSCLC cells by inhibiting the Nrf2/HO-1 singnaling pathway.

## Discussion

Natural active compounds extracted from herbs provide an inexhaustible pool of cancer therapy candidates *via* different mechanisms (Desai et al., 2008). It is generally known that inducing tumor cell apoptosis has long been identified as the primary anticancer mechanism of these products. However, an increasing amount of research suggests that numerous non-apoptotic patterns of PCD can be generated when the apoptosis process seems to be changed or inhibited (Indran et al., 2011; Giussani et al., 2014). Recently identified regulated cell death known as ferroptosis has been linked to several diseases, including cancer (Dixon et al., 2012). Due to the fact that cancer cells demand more iron than normal cells, this situation renders cancer cells more prone to ferroptosis (Hassannia et al., 2019). Therefore, promoting cancer cell ferroptosis may be a novel and effective cancer treatment strategy. ShtIX is a novel isoflavane compound from the heartwood of *D. odorifera* T. Chen, the anticancer activities and the underlying mechanism are still being investigated. Here, we found that ShtIX exhibited the highest cytotoxicity to NSCLC cells among the selected cancer cell lines.

In the present study, a variety of experimental methods were employed to demonstrate that ShtIX may induce NSCLC cell death but not in normal cells. Furthermore, the animal experiments also determined that ShtIX retarded tumor growth in xenografted nude mice. However, ShtIX only induces cell apoptosis at a dosage of 16 μM (double of IC50 value) according to the results of the apoptotic event test, inferring that apoptosis is not the primary mechanism of ShtIX-induced NSCLC cell death. In addition, apoptotic, necrosis and autophagy inhibitors were found to be ineffective in reversing ShtIX-induced NSCLC cell death. Contrarily, ShtIX-induced cell death in NSCLC cells could be neutralized by ferroptosis inhibitors, suggesting that an iron-dependent cell death pathway was involved in ShtIX-induced NSCLC cell death.

Iron ion is a crucial trace element in the human body that helps cells conducts a variety of biological processes. When iron homeostasis is disrupted, excess reactive oxygen species (ROS) are produced and leads to ferroptosis (Ying et al., 2021). Ferritin, which composed of ferritin heavy chain (FTH) and ferritin light chain (FTL), is a key protein in iron homeostasis. Abnormal FTH expression causes iron storage dysfunction and cell death by compromising cellular antioxidant defenses (Andrews, 2010). In the current study, the expression of FTH1 was markedly down-regulated after ShtIX treatment, implying that ShtIX can cause iron overload via reducing iron storage. Iron overload is a prerequisite for the occurrence of ferroptosis. Excessive iron in the cell can enhance the accumulation of intracellular ROS through Fenton reaction, which causes lipid peroxidation and initiates ferroptosis (Dixon et al., 2012). Here, our findings indicated that the ShtIX-induced ferroptosis was brought on by excessive lipid peroxidation as seen by the generation of excess Fe^2+^, accumulation of ROS and lipid ROS, and an increase in MDA, all of which were neutralized by ferroptosis inhibitor Fer-1. Additionally, GSH is essential for amino acid metabolism during ferroptosis and is crucial for maintaining cellular redox homeostasis. If GSH levels depleted, the cellular antioxidant defense mechanism would be disrupted, and ferroptosis would be triggered (Ursini and Maiorino, 2020). The intracellular GSH was obviously decreased with the concentration increases of ShtIX in this study. GSH deficiency can make GPX4 to become inactive and lead to ferroptosis. The “gatekeeper” of ferroptosis, GPX4, is a glutathione peroxidase that uses reduced GSH as a cofactor to get rid of lipid peroxidation. Inhibition of GPX4 would result in a buildup of lipid peroxidation, which would result in the occurrence of ferroptosis (Yang et al., 2014). Similarly, the activity of GPX4 decreased after cells were exposed to ShtIX, and the ferroptosis inhibitor fer-1 reversed this effect, demonstrating that ShtIX induced ferroptosis in NSCLC cells.

Numerous signaling pathways are involved in the mediation of ferroptosis in addition to those distinctive ferroptotic markers. Recently, a growing number of studies have revealed that the Nrf2/HO-1 signaling pathway can act as a negative regulator of ferroptosis (Jiang et al., 2020b; Ma et al., 2020; Li et al., 2021; Lou et al., 2021; Wei et al., 2021). The transcription factor Nrf2, which is crucial for regulating oxidative stress in cells, has been discovered to be constitutively activated or overexpressed in different cancer types. A variety of genes necessary for ferroptosis can be regulated by Nrf2 activation, including those that inhibit iron absorption, reduce ROS generation, and boost cellular antioxidant capability (Anandhan et al., 2020). Furthermore, it has been discovered that Nrf2 regulate a number of ROS-detoxifying enzymes, including HO-1 (Sajadimajd and Khazaei, 2018). In this study, the expression of Nrf2 and HO-1 in NSCLC cells was decreased with increasing concentration of ShtIX. Moreover, knocking down Nrf2 could effectively reduce the level of Nrf2 and HO-1 and strengthen ferroptosis induced by ShtIX, implying that ShtIX can promote ferroptosis by inhibiting Nrf2/HO-1 signaling pathway in NSCLC cells. In a xenograft mouse model, the anticancer effectiveness of ShtIX and the role of Nrf2/HO-1 in ShtIX-induced ferroptosis were also demonstrated.

**FIGURE 8:**
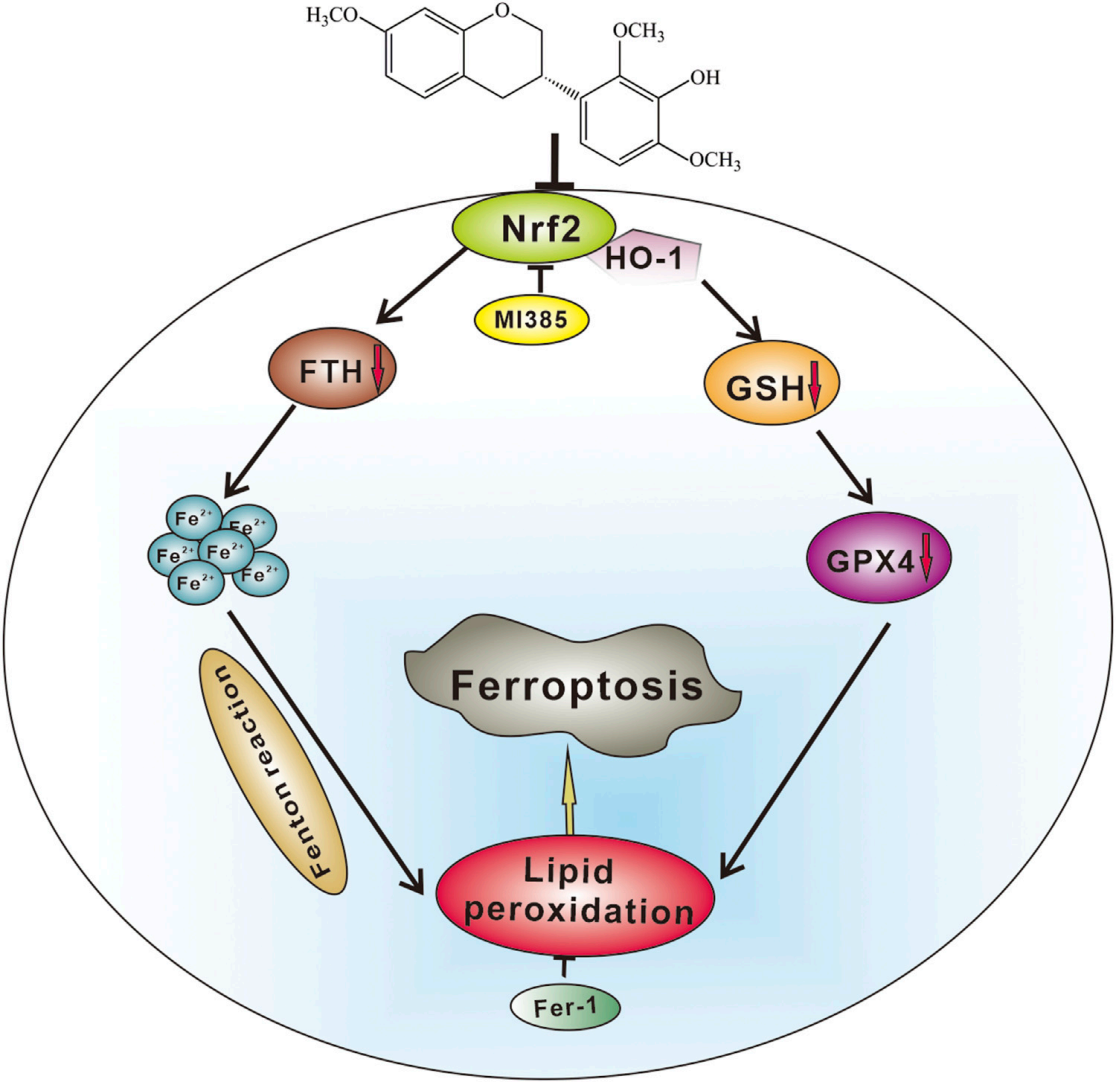
A hypothetical molecular mechanisms of ShtIX-induced ferroptosis in NSCLC cells. ShtIX induces NSCLC cells ferroptosis by inhibiting Nrf2/HO-1 signaling pathway.

In conclusion, the present study is the first to demonstrate that ShtIX causes NSCLC cell death *via* ferroptosis. ShtIX is able to disrupt iron homeostasis, increase Fe^2+^ counts, lower GSH levels, decrease GPX4 expression, and promote the buildup of lipid peroxides. Furthermore, the Nrf2/HO-1 signaling pathway was found to be involved in ShtIX-induced ferroptosis, and inhibiting the Nrf2/HO-1 pathway *in vitro* and *in vivo* can considerably enhance the effect of ShtIX-induced ferroptosis. The potential molecular mechanism of ShtIX-induced NSCLC cells ferroptosis is exhibited in Figure 8. The work establishes ShtIX as a potential natural ferroptosis inducer for the treatment of NSCLC.

## Data Availability

The original contributions presented in the study are included in the article/Supplementary material, further inquiries can be directed to the corresponding author.
